# Scanning Electron Microscopy of the Antennal Sensilla and Their Secretion Analysis in Adults of *Aromia bungii* (Faldermann, 1835) (Coleoptera, Cerambycidae)

**DOI:** 10.3390/insects10040088

**Published:** 2019-03-28

**Authors:** Antonella Di Palma, Marco Pistillo, Raffaele Griffo, Antonio P. Garonna, Giacinto S. Germinara

**Affiliations:** 1 Department of the Science of Agriculture Food and Environment, University of Foggia, Via Napoli 25, 71100 Foggia, Italy; marco.pistillo@unifg.it (M.P.); giacinto.germinara@unifg.it (G.S.G.); 2 Plant Protection Service of Campania Region, Centro Direzionale, Isola A6, 80124 Naples, Italy; r.griffo@maildip.regione.campania.it; 3 Department of Agriculture, University of Naples Federico II, Via Università 100, 80055 Portici, Italy; garonna@unina.it

**Keywords:** red-necked longhorn, chemoreceptors, mechanoreceptors, antennal secretions, contact pheromone, GC-MS

## Abstract

Background: It has been proved that chemical signals play an important role in mating location and reproductive behavior in cerambycids; moreover, they rely on contact chemoreception for mate recognition. Methods: Adult antennae of *Aromia bungii* were observed using scanning electron microscopy and adult antennal secretions were collected and analyzed with gas chromatography-mass spectrometry. Results: Twelve different types of sensilla were morphologically described on the antennae of *A. bungii*. At least six mechanoreceptors—one gustative, one putative chemo- or thermoreceptor, and three multiporous olfactory receptors—are present on the antennae of both sexes while a receptor-type of unclear function is limited to males. Secretions associated with sensilla basiconica were observed for the first time in a cerambycid species. Conclusions: Sensilla basiconica should play a role in odor perception detecting host tree volatiles and/or pheromones. Sensilla basiconica type 1 and 2 produce a viscous material accumulating on the antennal surface. Chemical analysis of adult antennal secretions highlighted marked differences between sexes. Some of the identified compounds have been previously reported as contact pheromone components of other cerambycid species. Our observations strongly suggest sensilla basiconica as the production sites of compounds involved in mate recognition.

## 1. Introduction

The red-necked longhorned beetle, *Aromia bungii* (Faldermann, 1835), is a wood-boring beetle and a major pest of stone fruit trees; it is native to the southeastern Palaearctic ecozone with an expansion in the Oriental Region, Europe and North America around 2008 [1,2]. In Europe, it was reported for the first time in field in Germany (Bavaria) on *Prunus domestica* subsp. *institia* (L.) Bonnier & Layens [3,4,5] and in Italy (Campania and Lombardia) on *Prunus armeniaca* (L.)*, P. avium* (L) L., *P. domestica* L., *P. persica* (L.) Batsch and *P. cerasifera* Ehrh. 1784 [6,7,8,9,10]. In these areas, it is currently under eradication. Moreover, in 2014, *A. bungii* was added to the EPPO A1 list of pests recommended to regulate as a quarantine pest [10,11]. Recently, the EU established specific restrictive measures to avoid introduction and diffusion of *A. bungii* in Europe [12].

The olfactory system is the primary sense that insects use in analyzing the environment in crucial tasks such as finding food and nesting, migrating, mating, oviposition, identifying conspecific, etc. [13]. Regarding cerambycids, although their chemical ecology has been little studied, it has been proved that chemical signals play an important role in mating location and reproductive behavior [14,15,16,17,18,19,20,21,22,23,24]. Moreover, longhorn beetles appear to rely on contact chemoreception for mate recognition. In fact, for several cerambycid beetles [15,19,25,26,27,28,29,30,31,32,33,34,35,36,37,38,39,40,41,42,43], antennae or palpi contact is necessary in mating recognition and, hence, required before mating takes place. In addition, in few cerambycid species, the presence of antennal glands, probably involved in sex recognition, have been reported [44,45]. However, to our knowledge, no study has yet been carried out to characterize the composition of the antennal gland secretions in the family Cerambycidae.

Thus, the purpose of this study was to describe types, morphology and distribution of the antennal sensilla in adults of *A. bungii*, using scanning electron microcopy (SEM), and to characterize the composition of the male and female antennal secretions by gas chromatography–mass spectrometry (GC-MS). This study might be useful to increase the biological knowledge on this beetle and clarify some reproductive aspects such as the role of antennation for sex communication and the involvement of tactile together with chemical stimuli. These are fundamental aspects to develop suitable monitoring and control tools of this economically important pest also considering its recent invasion of North America, Japan, and Europe and the likely invasion of additional countries.

## 2. Materials and Methods

### 2.1. Beetles

Logs containing *A. bungii* larvae were collected from cut down trees in the Marigliano (Naples, Campania, Italy) area on 11 April 2017 and placed in rearing cages to obtain the adults starting from the first week of June 2017. Newly emerged adults were collected daily and placed individually in transparent plastic containers (6 cm diameter × 8 cm height). To allow air exchange, the containers were covered with screw caps provided with a central hole (2 cm) screened by a metallic net (mesh size 1 mm). Insects were maintained at 25 ± 2 °C, 60 ± 5% relative humidity, and 16:8 Light:Dark photoperiod and fed ad libitum with apple pieces that were renewed every 3 days.

### 2.2. Scanning Electron Microscopy (SEM)

Individuals for scanning electron microscopy were sexed and the heads, carrying the antennae, were removed under a stereomicroscope, stored in 70% ethanol and subsequently dehydrated in a graded alcohol series of 80%, 90%, and 100% ethanol. Then, the antennae were mounted on a stub with double-sided adhesive tape and gold sputtered in a Baltec SCD 005 sputter coater. The antennae were mounted on dorsal and ventral sides (both male and female usually keep the antennae extended in front of the head so that the general downward facing curvature of the antennae is retained and a dorsal and ventral side can be easily distinguished) on the stubs and examined and micrographed with a Hitachi TM3030 scanning electron microscope.

### 2.3. Preparation of Antennal Secretion Extracts

Antennal secretions present on the surface of the sixth to the ninth flagellomeres of 4-week-old living males and females were gently removed using the tip of sterile syringe needles paying attention to avoid antennal breaking and hemolymph leakage. Antennal secretions (about 0.5 mg) collected from specimens (*n* = 3) of each sex were dissolved in n-hexane (500 µL) and stored at −20 °C until needed.

### 2.4. Gas Chromatography-Mass Spectrometry (GC-MS)

One microliter of extract was analyzed by a 7890B series gas chromatograph (Agilent Technologies) coupled with an Agilent 5977A mass selective detector (MSD) and equipped with a HP-5MS capillary column (30 mm × 0.25 mm i.d., × 0.5 µm film thickness, J&W Scientific Inc., Folsom, CA, USA). The carrier gas was helium at a flow rate of 1.25 mL/min. The injection was made in the splitless mode, and the injector temperature was 250 °C. The column oven temperature was initially programmed from 100 °C to 300 °C at 10 °C/min, with a final holding time of 15 min. Spectra were recorded in the electron impact mode (ionization energy, 70 eV) in a range of 15–550 amu at 2.9 scans/s. A solvent delay time of 5 min was used. Each extract was analyzed in triplicate. Solvent controls were analyzed to check for interferences.

Straight-chained saturated alkanes were identified by their molecular ions and from comparisons of retention times and mass spectra with those of authentic standards. Methyl branched compounds were identified from their Kovats retention indices relative to straight chain hydrocarbons [46] in combination with diagnostic ions from enhanced fragmentations of methyl branch [47,48,49]. Unsaturated alkenes were identified by molecular weight, from retention times slightly shorter than those of the corresponding straight chain saturated alkanes, and their characteristic patterns of ions with masses two or four mass units less than the corresponding ions in the spectra of straight-chain alkanes, for monoenes and dienes, respectively [50].

Besides, comparison of MS fragmentation patterns with those included in the National Institute for Standards and Technology database (NIST 02, *p* > 80) were utilized to support tentative identification. The relative abundance of each compounds was calculated using the integrated peak area data from the GC-MS trace.

## 3. Results and Discussion

### 3.1. Antennal General Morphology

**Figure 1:**
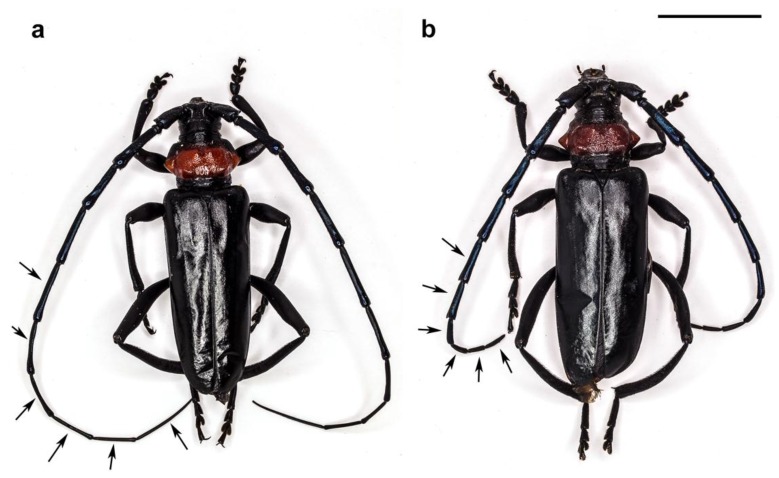
*A. bungii*, LM general overview: male (**a**) and female (**b**). Arrows point to the 4th–9th flagellomeres to show length differences between sexes. Scale bar: 1 cm.

**Figure 2:**
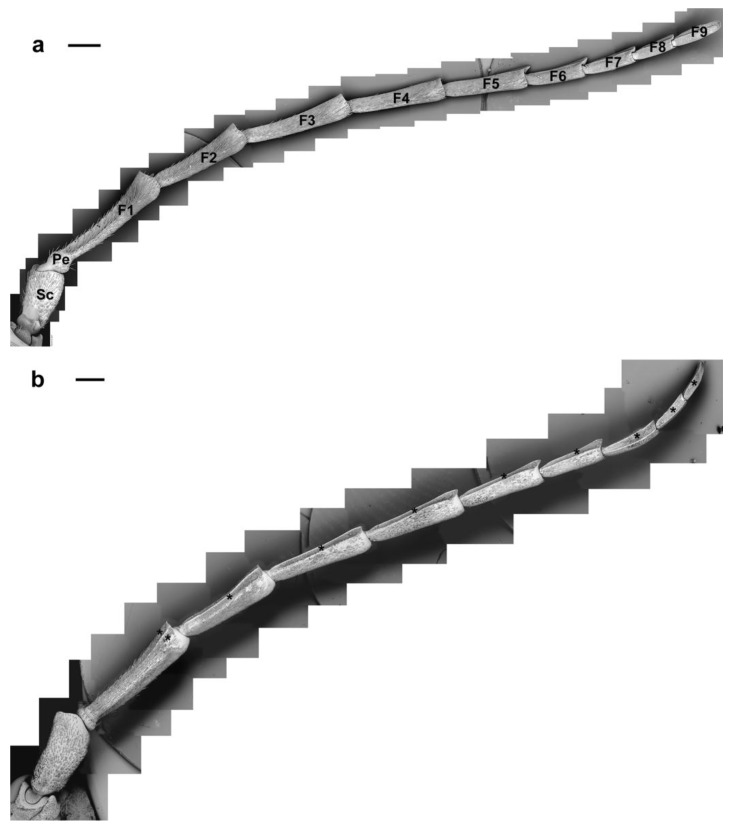
*A. bungii*, SEM. Overview of the female antennae: (**a**) dorsal view, left antenna; and (**b**) ventral view, left antenna (asterisks indicate the flat longitudinal bands facing the abaxial surface). Abbreviations: F1–F9, flagellomere 1st–9th; Pe, pedicel; Sc, scape; Scale bars, 1 mm.

In both sexes, the antennae are morphologically similar: filiform, consisting of two basal segments (the scape and a short pedicel) and a long flagellum composed of nine flagellomeres (Figure 1 and Figure 2). Males present much longer antennae (mean length 56.4 ± 1.9 mm) than the females (mean length 30.1 ± 1.86 mm) due to the differences in the length from the fourth to the ninth flagellomere (Figure 1, arrows).

Each antenna presents two flat longitudinal bands, one on the ventral and one on the dorsal side, of variable size and facing the abaxial surface (Figure 2b, asterisks). Such bands are easily visible since they are delimitated by longitudinal strips devoid of any sensilla (Figure 2b asterisks). Six types of sensilla chaetica (SC1–6), one type of sensilla trichodea (ST1), four types of sensilla basiconica (SB1–4) and Böhm bristles (BB) were distinguished on the antennae on the base of their external morphology from SEM observations (according to Schneider [51]) (Table 1).

Where not indicated otherwise, type and distribution of the sensilla refer to both male and female.

### 3.2. Antennal Sensilla and Their Secretion Analysis

#### 3.2.1. Sensilla Chaetica

They are the most common sensilla on the antennae and consist of a long hair-shaft set in an obvious flexible socket. The outer surface is sculptured by dense longitudinal grooves. Several types are distinguishable according to their hair-shaft size, shape of grooves, and their location. Most aporous sensilla chaetica with a flexible socket (SC1, SC2, SC4, SC5, and SC6) could be assumed to be mechanoreceptors involved in different kinds of activities.

**Figure 3:**
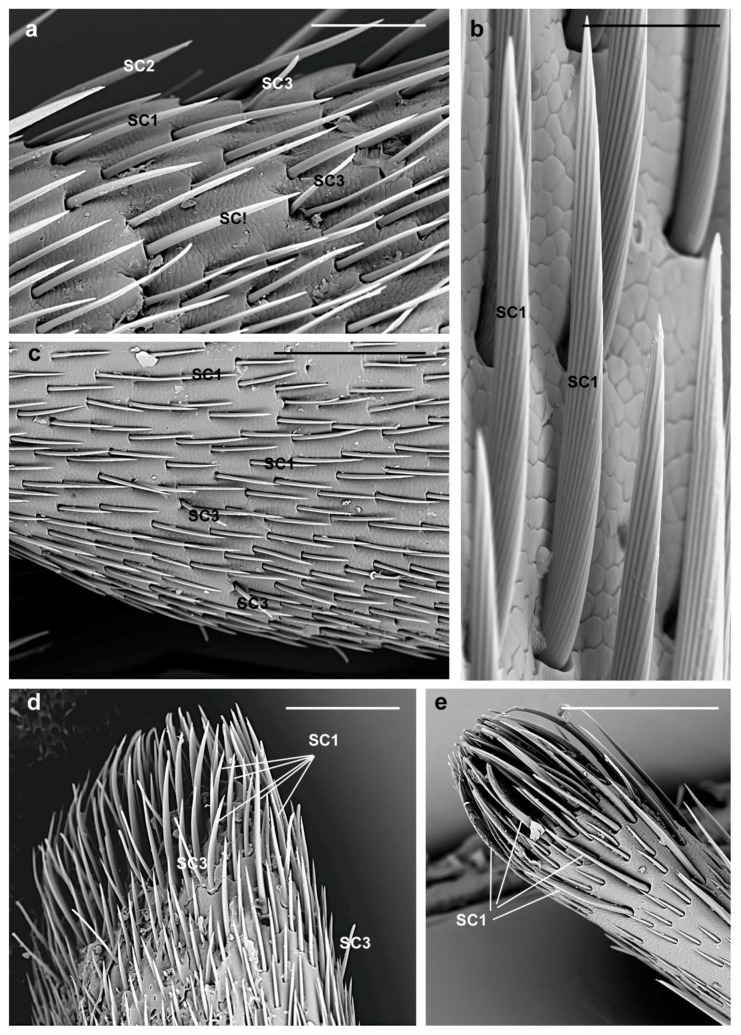
*A. bungii*, SEM. Sensilla chaetica SC1. (**a**) Overview of SC1 on the female second flagellomere. (**b**) Detail of the V-shaped grooved pattern on the shaft surface and the sharp tip. (**c**) View of the even distribution around the circumference of the female antennomeres. SC1 on the tip of ninth flagellomere in female (**d**) and male (**e**). Abbreviations: SC1, sensillum chaeticum type 1; SC2, sensillum chaeticum type 2; SC3, sensillum chaeticum type 3. Scale bars: 100 µm (**a**,**d**,**e**); 30 µm (**b**); and 200 µm (C).

**SC1.** These sensilla are slightly curved and lay parallel to the antennal surface pointing towards its tip (Figure 3a,c); they have a V-shaped deep grooved pattern on the shaft surface that ends in a sharp tip (Figure 3b). Their length (female 95.8 ± 39.9 µm; male 99.9 ± 28.8 µm) can be variable: from 45 µm (those present in the longitudinal bands of the antennae and covering sensilla basiconica), 120–130 µm (those elsewhere on the surface of the antennomeres) to 140 µm at the tip of the ninth flagellomere (Figure 3d,e). They are evenly distributed around the circumference of each antennomere (Figure 3a,c), except for a glabrous region devoid of any sensilla (Figure 2b), and cover the underlying sensilla basiconica. Thus, they may inform the insect of the different antennal contacts and protect the underlying basiconica.

**Figure 4:**
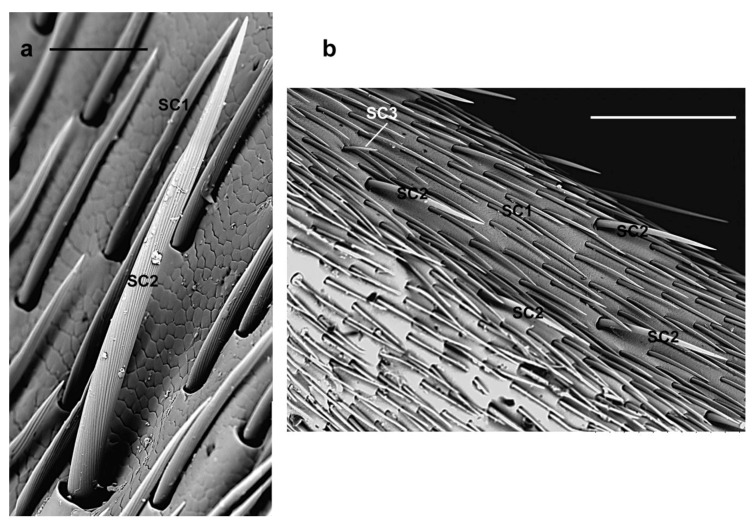
*A. bungii*, male, SEM. Sensilla chaetica SC2. (**a**) Detail of the grooved bristle with articulated socket. (**b**) Overview of the third flagellomere showing SC2 easily distinguished from SC1 by their longer shaft slightly diverging from the antennal surface. Abbreviations: SC1, sensillum chaeticum type 1; SC2, sensillum chaeticum type 2; SC3, sensillum chaeticum type 3. Scale bars: 50 µm (**a**); and 300 µm (**b**).

**SC2.** They have longitudinally grooved bristles (female 233.9 ± 35.4 µm; male 225.2 ± 51.6 µm), are slightly tapered with pointed tips, located in an open articulating socket, and pointing toward the tip of the antennae (Figure 4a). They are distributed around the circumference of scape, pedicel and flagellomeres and easily distinguished from SC1 since they are longer and their straight shaft is slightly diverging from the antennal surface (Figure 3a and Figure 4b). Hence, they might be the first to contact the substrate during antennation and act as mechanoreceptors helping the insect in directing the antennae.

**Figure 5:**
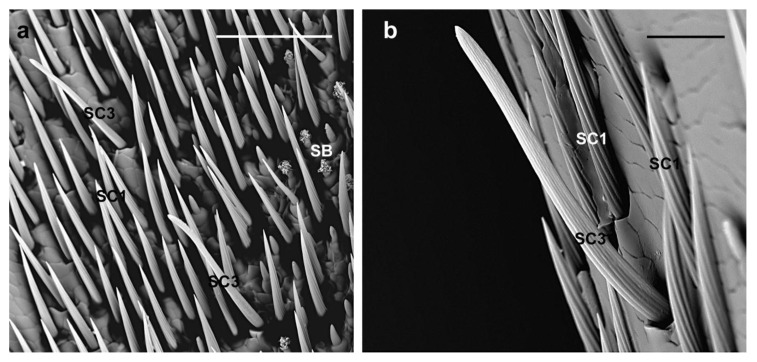
*A. bungii*, SEM. Sensilla chaetica SC3. (**a**) Female: Two SC3 with several SC1 and some SB (different types). (**b**) Male: Detail of one SC3 with thin parallel grooves and blunt tip (compared to SC1). SC1, sensillum chaeticum type 1; SC3, sensillum chaeticum type 3; SB, sensilla basiconica. Scale bars: 50 µm (**a**); and 30 µm (**b**).

**SC3.** They are straight with a shaft raised from the antennal surface at an angle of about 45° and easily visible since they appear electron lucent (Figure 3a,c,d and Figure 5a). These sensilla present thin parallel grooves along their length (female 68.9 ± 9.5 µm; male 67.9 ± 15 µm) and blunt tips (compared to SC1) (Figure 5b). Considering their arrangement around the circumference of the antenna (Figure 3a,c), and among the distal tuft of setae (Figure 3d), the flexible socket, the shaft raised from the antenna surface and the blunt tip, they resemble uniporous gustatory sensilla acting as multimodal [52,53]. In fact, they might serve as contact chemoreceptors and could well be used to sense specific cuticular hydrocarbons, hence playing a role in mate recognition during courtship behavior. In fact, contact chemoreception has been shown to play an important role in mating behavior for several cerambycid species [15,21,25,26,27,54]. SC3 may also be sensitive to host plant cues since adults of both sexes regularly tap the surface of the substrate they are currently on.

**Figure 6:**
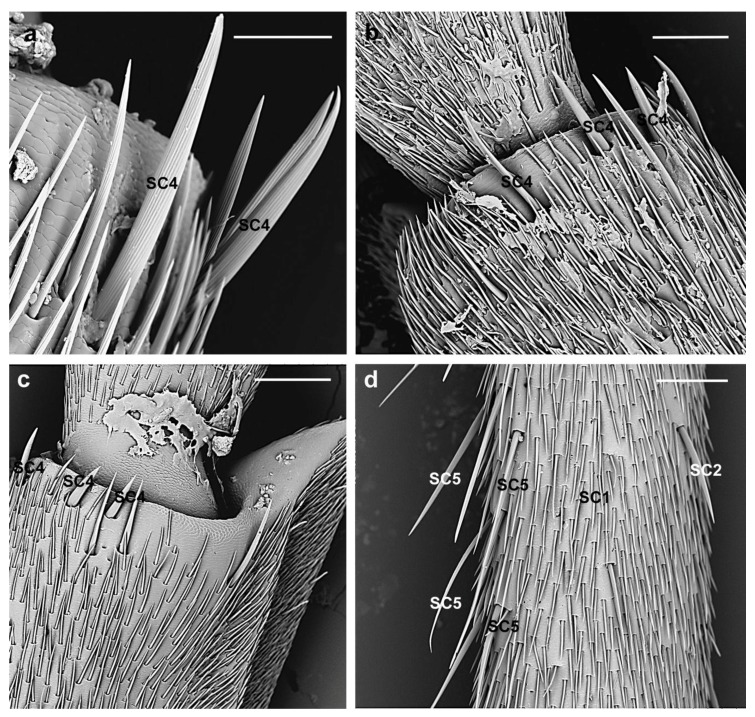
*A. bungii* female, SEM. Sensilla chaetica SC4 and SC5. (**a**) Details of SC4: Articulated bristles, with longitudinal grooves. (**b**,**c**) Several SC4 arranged in horizontal line on the distal region of the flagellomeres. (**d**) SC5 provided with long, straight, grooved shafts. Abbreviations: SC1, sensillum chaeticum type 1; SC2, sensillum chaeticum type 2; SC4, sensillum chaeticum type 4; SC5, sensillum chaeticum type 5. Scale bars: 50 µm (**a**); 100 µm (**b**); and 150 µm (**c**,**d**).

**SC4.** These sensilla are very similar in appearance to SC1 but much longer (female 209.4 ± 50.6 µm; male 277.3 ± 84 µm). They are large, articulated bristles, with longitudinal grooves accumulating toward the tip (Figure 6a). They lay parallel to the surface pointing toward the tip of the antennae, are absent from the scape and pedicel, and occur on the distal region of the flagellomeres arranged in horizontal line (Figure 6b,c). Thus, they might act as proprioceptors stimulated by the change in the position of the adjacent segments (movements of the flagellomeres) or when the antennae are bent too far inwards and, hence, help retain the curved shape of the antennae.

**SC5.** These sensilla are very long (female 329.4 ± 35.2 µm; male 384.4 ± 82.3 µm) straight, stout with a grooved shaft (Figure 6d). They are absent from the scape and pedicel, while present in the 1st–6th flagellomere on the antennal dorsal side only. Considering their location, these sensilla might be involved as mechanoreceptors in the antennation observed in many cerambycid species during mating.

**Figure 7:**
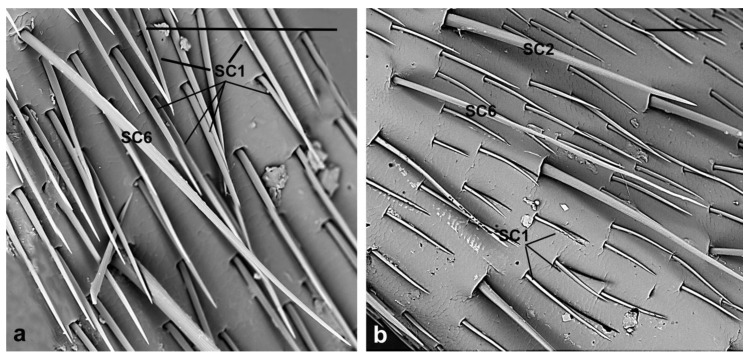
*A. bungii*, SEM. Sensilla chaetica SC6. (**a**) Female: Detail of one SC6 with a very long, thin shaft provided with longitudinal grooves and pointed tips. (**b**) SC6 in male. Abbreviations: SC1, sensillum chaeticum type 1; SC2, sensillum chaeticum type 2; SC6, sensillum chaeticum type 6. Scale bars: 100 µm.

**SC6.** These sensilla have a very long shaft (female 367 ± 53.2 µm; male 411 ± 68.3 µm), thin, irregularly curved with longitudinal grooves and pointed tips (Figure 7). They are present only on the dorsal side of the flagellomeres 1st–5th in number of two-four, often arranged in a straight longitudinal line. They might respond to sounds or air currents, as suggested also by Dyer and Seabrook [44] and Faucheux [45]. *Aromia* is, in fact, known to be able to stridulate (personal observations), so such setae can work as sound as well as wind receptors.

**Figure 8:**
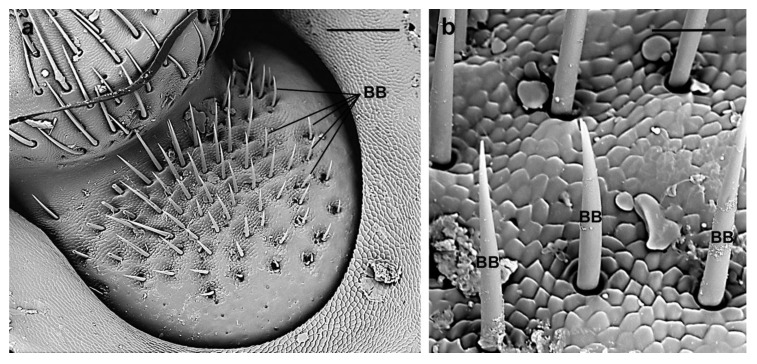
*A. bungii* female antenna at the level between head and scape, SEM. (**a**) Böhm bristles: Spine-shaped sensilla inserted into well-developed cuticular sockets. (**b**) Detail of the sharp tips and smooth cuticle. Abbreviations: BB, Böhm bristle. Scale bars: 150 µm (**a**); and 25 µm (**b**).

**Böhm bristle (BB).** These are straight, or slightly curved, spine-shaped sensilla (female 64.8 ± 17 µm; male 62.5 ± 13 µm) inserted into well-developed cuticular sockets and showing sharp tips and smooth cuticle (Figure 8). According to their concentration in a dense group on the inter-segmental joints between the scape and the head (Figure 8a), as well as between the scape and the pedicel, and their wide articulated socket, they likely function as proprioceptors informing the insect of the antennal position and movements, as in many other groups of insects [51].

#### 3.2.2. Sensilla Trichodea

They are much less common than the chaetica and characterized by a hair-shaft without a discrete membranous socket distinguishable at SEM.

**Figure 9:**
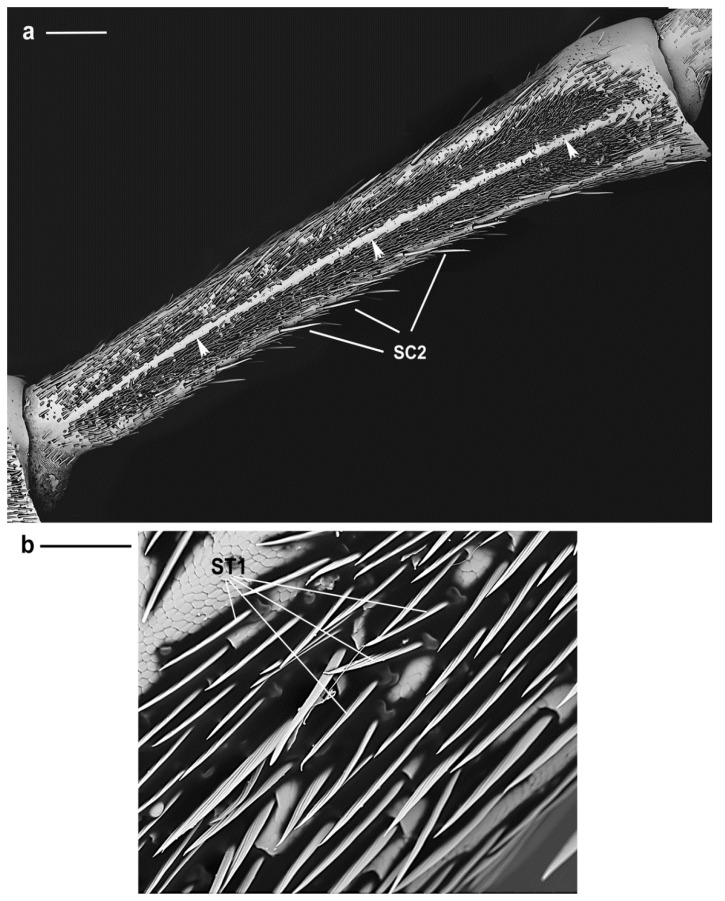
*A. bungii* male, SEM. (**a**) Overview of the second flagellomere with the two longitudinal bands facing the abaxial surface and separated by a longitudinal strip devoid of any sensilla (arrowheads). Such bands are packed with SC1, SC2, SB and ST1. (**b**) Detail of ST1 showing a thin straight shaft projecting from a raised base with no evident articulation. Abbreviations: SC2, sensillum chaeticum type 2; ST1, sensillum trichodeum type 1. Scale bars: 500 µm (**a**); and 100 µm (**b**).

**ST1.** These sensilla are thin with a grooved straight shaft (57.5 ± 4.4 µm long) parallel to the antenna and projecting from a raised base (Figure 9) with no evident articulation. They only occur in males and are located in the abaxial longitudinal bands of the first and second flagellomeres where they are closely packed with SC1, SC3, few SC2, and with sensilla basiconica (SB) (Figure 9a,b). On the first flagellomere ST1 are present only in the distal part of the bands. It was not possible to ascertain if their walls are provided with pores, while their base, hardly visible because covered by SC1, seems not provided with a flexible socket. Their function remains unclear.

#### 3.2.3. Sensilla Basiconica

**Figure 10:**
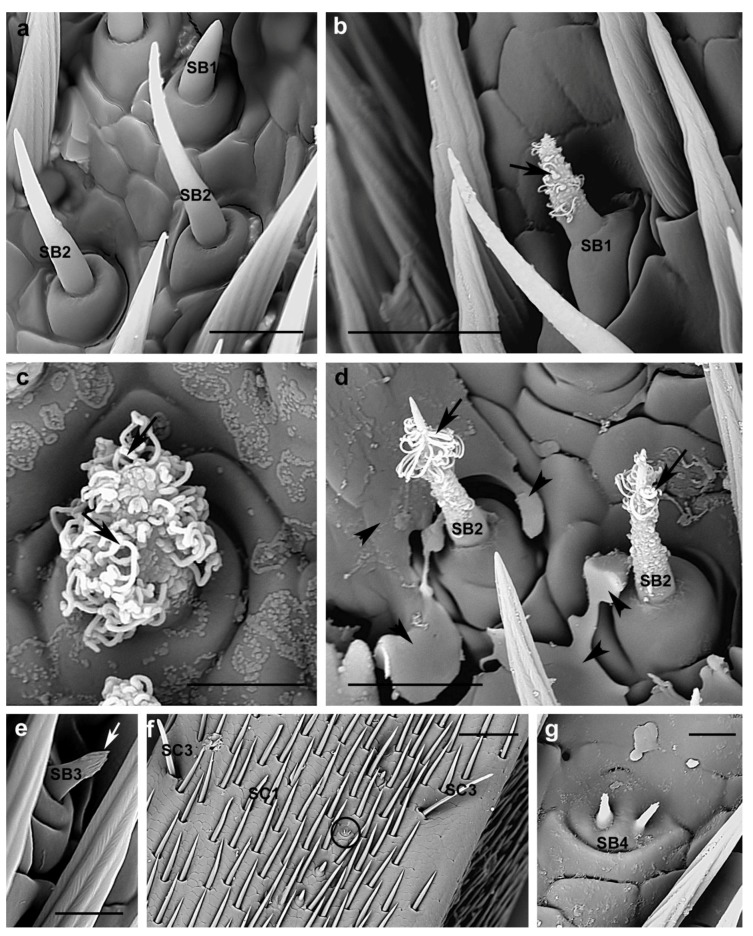
*A. bungii*, SEM. Sensilla basiconica. (**a**) Female: SB1 and SB2. (**b**) Detail of one SB1 covered by abundant viscous material condensing outside in female and (**c**) male (arrows). (**d**) Male: Detail of two SB2 with some viscous material condensing outside their walls (arrows) and on the antennal surface (arrowheads). (**e**) Male: Detail of one SB3 with smooth base and distal finger-like projections (arrow). (**f**) Male: Overview of one SB4 (circle) visible among several SC1 and few SB2. (**g**) Male: Detail of SB4 represented by two jointed sensilla raising from the same base. Abbreviations: SB1, sensillum basiconicum type 1; SB2, sensillum basiconicum type 2; SB3, sensillum basiconicum type 3; SB4, sensillum basiconicum type 4; SC1, sensillum chaeticum type 1; SC3, sensillum chaeticum type 3. Scale bars: 10 µm (**a**,**b**,**d**); 5 µm (**c**,**e**,**g**); and 50 µm (**f**).

**SB1.** They are blunt-tipped, curved, relatively short sensilla (female 9.5 ± 1 µm; male 8.5 ± 0.7 µm). They present a smooth shaft, without longitudinal grooves, projecting from an elevated base without articulating socket (Figure 10a). They appear, sometimes, covered by abundant viscous material clogging the pores and condensing outside (Figure 10b,c).

**SB2.** These sensilla are thinner and longer than SB1 (female 20.3 ± 3.8 µm; male 18.1 ±3.3 µm); they have a sharper tip, a bent shaft emerging from an elevated socket, and smooth cuticle (Figure 10a). Sometimes they present some viscous material condensing outside their walls (Figure 10d).

Both in SB1 and SB2, detection of pores was highlighted by the thread-like secretion extruded by their walls.

**SB3 (grooved peg sensilla).** These sensilla are very short (female 5.9 ± 0.8 µm; male 5.3 ± 0.8 µm), smooth at the base with the distal half surrounded by finger-like projections (Figure 10e, arrow) tapering gradually to a blunt tip; they insert into a wide dome and present no articulating socket (Figure 10e). These sensilla are rare (three-six per article), in both males and females, and scattered among the other basiconic sensilla (SB1 and SB2) and sensilla chaetica SC1 and SC3.

**SB4.** They are short jointed sensilla raising from the same base, represented by a flattened wide dome, with no evident socket; they show a smooth shaft with blunt tip (Figure 10f,g). These sensilla were observed in males and females but, in both cases, just one sensillum was found located on the ventral side of the ninth flagellomere among other SB1 and 2 and SC1 (Figure 10f).

**Figure 11:**
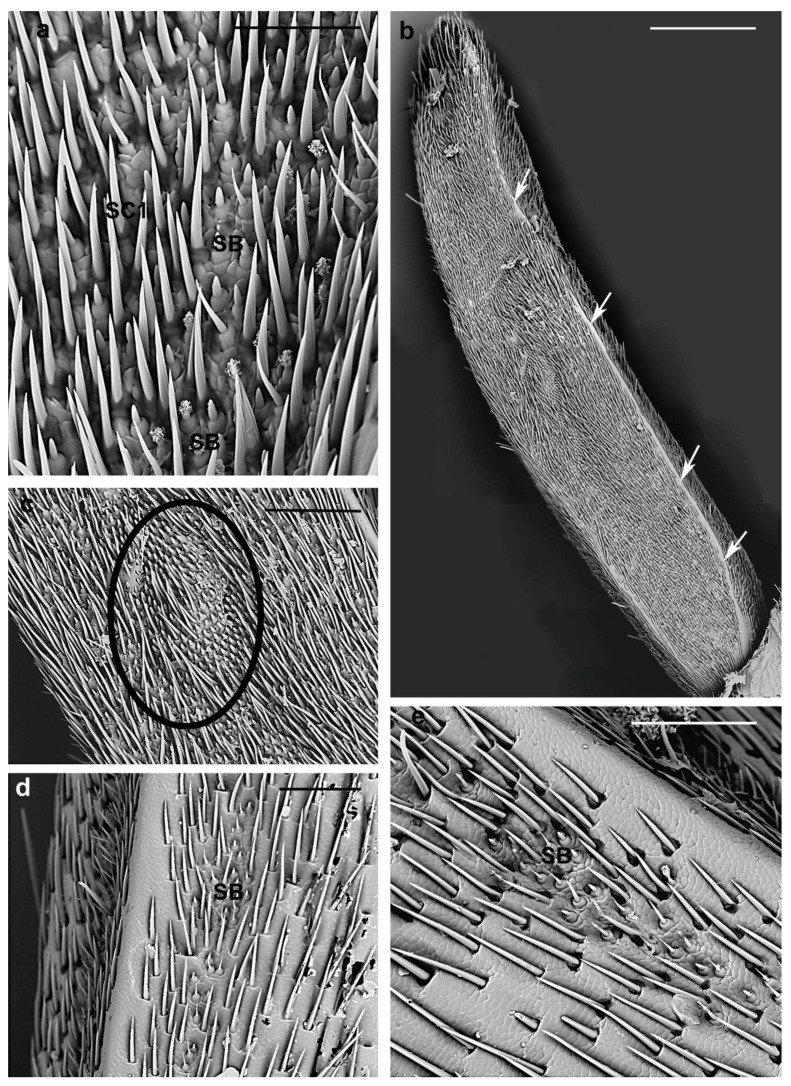
*A. bungii* female, SEM. Sensilla basiconica. (**a**) SB1 and SB2 densely covered by closely packed SC1. (**b**) Ventro-dorsal view of the ninth flagellomere showing the two bands densely covered by SB, SC and separated by a glabrous strip (arrows). (**c**) Detail of the previous one showing SB organized in very gathered clusters (circle). (**d**,**e**) Variable concentration of SB along the longitudinal bands. Abbreviations: SB, sensillum basiconicum; SC1, sensillum chaeticum type 1. Scale bars: 50 µm (**a**); 300 µm (**b**); and 100 µm (**c**,**e**).

Sensilla basiconica SB1, SB2, and SB3 are present in all flagellomeres (thus absent in pedicel and scape), yet their distribution is patchy and they are mainly concentrated in the two lateral bands, one dorsal and one ventral, facing the abaxial surface of the antennae (Figure 2b and Figure 9a). In these zones, SB appear densely covered by a layer of closely packed SC1 (Figure 11a) together with a few SC3. Few basiconica are visible in the nearby zone close to these bands.

In females, these bands are thinner in the basal flagellomeres (1st–5th) while in the distal flagellomeres (7th–8th) they occupy almost half of the ventral and dorsal surface of the flagellomeres (Figure 2b) and in the ninth these bands cover the entire ventral and dorsal surface up to the distal tuft of setae (Figure 11b). Moreover, the distribution of SB inside these bands is patchy: the ninth flagellomere shows zones where SB are very dense and organized in gathered clusters (Figure 11b,c) with a peculiar distribution of SB1 and SB2, while in the other flagellomeres the concentration of SB is variable along the longitudinal bands but without clusters (Figure 11d,e).

**Figure 12:**
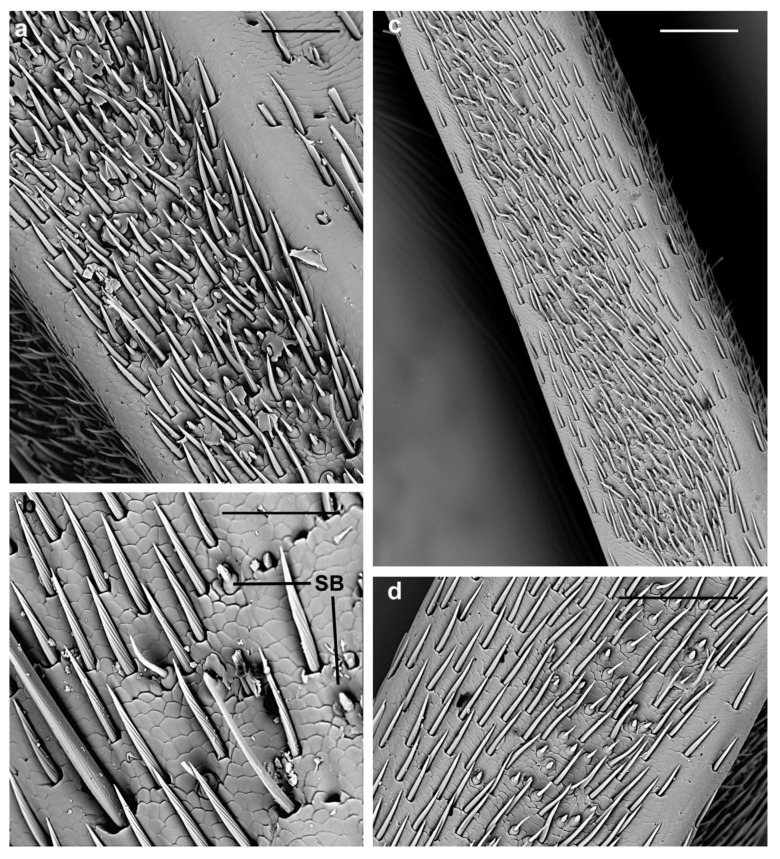
*A. bungii* male, SEM. Sensilla basiconica. (**a**) Numerous SB1 and SB2 concentrated in one lateral band. (**b**) Detail of few SB visible elsewhere outside the lateral bands. (**c**) Ventral view of the ninth flagellomere with a few basiconica not covering the entire surface as in females. (**d**) Overview of few SB in the basal region of a flagellomere showing a lower concentration compared with A. Abbreviations: SB, sensillum basiconicum. Scale bars: 50 µm (**a**,**c**); and 100 µm (**b**,**d**).

In males, SB are also concentrated in the two lateral bands (Figure 12a), with few basiconica visible elsewhere outside these strips (Figure 12b). These bands do not cover the entire ventral and dorsal surface of the ninth flagellomere (Figure 12c) as in females. Moreover, SB do not reach the distal tuft of setae (Figure 3e) and do not show a cluster organization (Figure 12c). Regarding their distribution, as in female, SB concentration is not uniform in the areas where they are present (compare Figure 12a,d).

Sensilla basiconica are typical multiporous olfactory receptors that respond to odors [53,55,56,57,58,59,60]. We therefore hypothesize that the flagellar sensilla basiconica SB1 and SB2 play a role in odor perception. The presence of different SB types on almost all the surface of the distal flagellomeres might indicate these are the main areas devoted to detect different chemical signals. Moreover, their concentration in the lateral longitudinal bands along the abaxial surface of the antennae seems to be designed for improving the catching efficiency of olfactory stimuli while the organization in cluster of variable size appears to be common in Coleoptera and cerambycid species [44,45,59,60,61,62,63,64,65,66]. It has been inferred that such olfactory sensilla clusters function as sensory fields representing an enlarged odor-sensing area that would be advantageous for long-distance olfactory detection [44,59,62]. In *A. bungii*, the sexual dimorphism of SB clustering (present only in female) might suggest that they are involved in detecting male sex pheromones, as suggested by Chen et al. [66] for males in *Xylotrechus grayii* (White, 1855).

Moreover, SB1 and SB2 present, quite often, in both males and females, an abundant and viscous thread-like secretion clogging the pores and condensing outside their walls; thus, these sensilla appear coated with solid curls or coiled ribbons (Figure 10b–d) suggesting the production of a secretion that appears to be non-volatile and accumulating on the antennal surface (Figure 10d, arrowheads).

In this respect, it is already reported the presence of glands associated with antennae and sensilla in Coleoptera and few other insect orders (e.g., [67,68,69,70,71,72,73,74,75,76,77,78]). In the family Cerambycidae, Dyer and Seabrook [44] observed that, in *Monochamus notatus* (Drury) and *M. scutellatus* (Say), the antennae are well supplied with dermal glands and flask-shaped glands opening near the base of different kind of sensilla. Moreover, in *Phoracantha recurva* (Newman, 1840), Faucheux [45] found holes associated with aporous sensilla, and Álvarez et al. [23], in another species of *Monochamus*, observed pore associated glands on the antennae. The possible function of the dermal glands on the antennae has been thought to be the secretion of the cement layer over the epicuticle after a molt while, for the glands associated with the sensilla, the secretion of cuticular hydrocarbons seems possible [23,44]. In addition, Dyer and Seabrook [44] and Faucheux [45] hypothesized that one of these two types of glands secretes a pheromone used in sex recognition. Nevertheless, as far as we know, secretions associated with sensilla basiconica have never previously been observed in any cerambycid species while they were observed once in pipunculid flies (Diptera) [78].

Remarkably, in few insect orders, it has been proved that several structures on the antennae are release sites of exocrine glands leading to the question if they are still sensilla or glands [69,74,75,78]. Moreover, antennation has been observed in several species in pre-copulatory and post-copulatory phase and, sometimes, correlated with the presence of peculiar antennal structures functioning as gland release sites. This discovery has profoundly changed the morpho-functional view of the antennae that can, hence, be involved in sex recognition, producing contact or volatile pheromones [69,70,72,79]. Behavioral studies of many species of long-horned beetles have confirmed that contact pheromones play an important role in reproduction: males appearing to recognize possible mates only after the antennae contact the antennae, or some other parts of the body, of the other conspecifics [15,25,26,27,28,29,30,31,32,33,34,35,36,37,38,39,40,41,42,43,44,80].

**Figure 13:**
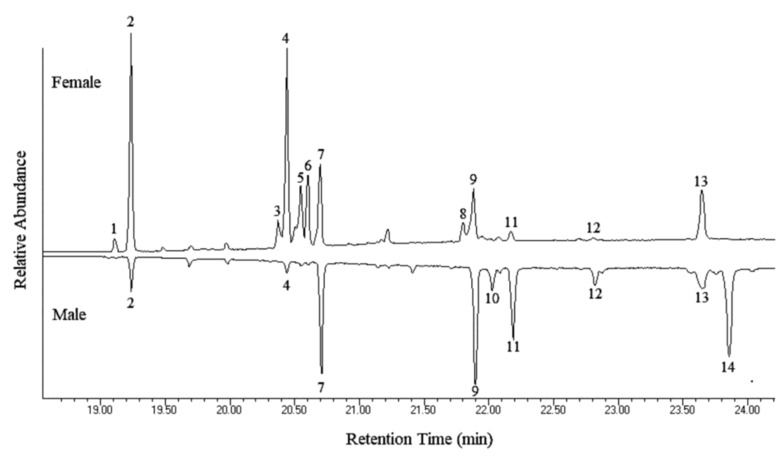
Gas chromatogram profiles of solvent extracts of *A. bungii* antennal secretions: female (top) versus male (bottom, inverted).

In this study, GC-MS analyses of the hexane extracts of male and female antennal secretions detected a total of 14 saturated and unsaturated aliphatic hydrocarbons, ranging in chain length from C_23_ to C_31_. Dominant compounds in the extract from males included C_27_ (Peak 7, Figure 13), 2-Me-C_28_ (Peak 9), C_29_ (Peak 11), and C_31_-monoene (Peak 14). The main components of the female extract were C_25_ (Peak 2), 2-Me-C_26_ (Peak 4), two C_27_-monoenes (Peaks 5 and 6), C_27_ (Peak 7), and 2-Me-C_30_ (Peak 13). Interestingly, some of these compounds have been previously reported as contact pheromone components of other cerambycid species [81]. Moreover, differences in the chemical composition of male and female antennal secretions were found. In fact, two compounds were specific to male, seven specific to female and seven in common to both sexes. Among the latter, remarkably, differences were found in the relative abundance of some compounds with the male extract having higher proportions of compounds with longer chain length (Figure 13 and Table 2), as observed in the longhorns *Xylotrechus colonus* (Fabricius, 1775) [30] and *Megacyllene caryae* (Gahan, 1908) [33]. Differences between the male and female cuticular hydrocarbon profiles have also been found in other cerambycid species and may result in different semiochemical functions ([82] and references therein). Based on this, one could speculate that, in *Aromia*, SB1 and SB2 might secrete a nonvolatile pheromone used for sex recognition.

According to Fukaya et al. [83], in *A. bungii*, male lure cages proved to be attractive to females that were induced to fly upwind by their presence. These results suggested that males release a long-range attractant signal received by females. Moreover, a male-produced sex-aggregation pheromone (*sensu* Cardé), was identified in *A. bungii* by Xu et al. [84]. Unfortunately, species within the tribe of Callichromini (subfamily Cerambycinae), including those of the genus *Aromia*, have not been sufficiently investigated in regard to their mating behavior. Thus, what we might assume is that males and females, as in other Cerambycidae [22,23,59], perceive host plant odors, and once on the host, males attract both sexes from some distance with aggregation pheromones and then recognize females by contact pheromones. If this is the case, antennae are not only receivers or various types of signals but also emitters of chemical messages and the sensilla basiconica (SB1 and SB2) might be the release sites.

Regarding SB3, they are grooved peg sensilla as observed in other beetle species and, based on their ultrastructure, their probable function is chemo- or thermoreception [53,58,60].

Finally, SB4 might represent a branched, extremely rare, variant of sensilla basiconica, as reported in *M. notatus,* by Dyer and Seabrook [44], and in *Tetropium fuscum* (Fabr.), by MacKay et al. [60]. They were interpreted as a morphological alteration due to stress during the development and hence not representing a distinct sensillar type. Nevertheless, it is worth noting that, in *T. fuscum*, such sensilla, as well as in *Aromia*, were observed only on the ninth flagellomere. Thus, it might be interesting to consider a single sensillum recording (SSR) to clarify functional aspects related to this sensillum type.

The external morphology of the antennal sensilla observed in *A. bungii* is similar to that described for other cerambycids [44,45,59,60,63,65,66,80] where sensilla chaetica, trichodea and basiconica are common, although with differences in the types; on the other hand, sensilla campaniformia and squamiformia have not been detected as opposed to Dai and Honda [63], Dyer and Seabrook [44] and Chen et al. [66].

To precisely ascertain the functions of the different sensilla, transmission electron microscopy (TEM) along with electrophysiological recordings must be conducted; moreover, single sensillum recording (SSR) will clarify the role of the different putative olfactory sensilla (SB1, SB2, SB3, and SB4) in processing pheromones, host, and non-host volatiles. Additionally, future ultrastructural observations with TEM, will likely confirm the presence of glands associated to basiconica sensilla in *Aromia* and help understanding the functional morphology of these glandular (?) sensilla. Moreover, behavioral bioassays are needed to clarify the biological activity of the compounds present in the male and female antennal secretions.

## 4. Conclusions

In our study, twelve different types of sensilla were morphologically described on the antennae of *A. bungii* using SEM. At least six mechanoreceptors—one gustative, one putative chemo- or thermoreceptor, and two multiporous olfactory receptors—are present on the antennae of both sexes while an additional receptor-type of unclear function is limited to males. From an olfactory perspective, the most interesting type of sensilla is the small basiconica playing a role in detecting volatiles. Moreover, secretions associated with sensilla basiconica were described for the first time in a cerambycid species. In particular, two types of sensilla basiconica produce a viscous thread-like material that accumulates on the antennal surface. The GC-MS analyses of the hexane extracts of the adult antennal secretions highlighted marked differences between sexes in the number and relative abundance of compounds. Moreover, since some of the identified compounds have been previously reported as contact pheromone components of other cerambycid species, SB1 and SB2 might the release sites of a nonvolatile pheromone used for mate recognition. This study provides a base for future investigations aiming at the development of semiochemical-based control means of this pest.

## Figures and Tables

**Table 1:** Types of sensilla observed on the antennae of *A. bungii*.

Sensillum Type	Location	Length	Shaft Aspect	Angle	Tips
**SC1**	Evenly distributed around the circumference of each antennomere	Variable from 45 µm to 140 µm	V-shaped grooves	Parallel to the antennal surface	Sharp
**SC2**	Around the circumference of scape, pedicel and flagellomeres	female 233.9 ± 35.4 µm; male 225.2 ± 51.6 µm	Longitudinal grooves	Shaft slightly diverging from the antennal surface	Tapering
**SC3**	Around the circumference of flagellomeres and among the distal tuft of setae	female 68.9 ± 9.5 µm; male 67.9 ± 15 µm	Thin parallel grooves	Shaft at an angle of about 45° on the antennal surface	Blunt
**SC4**	Arranged in a horizontal line on the distal region of the flagellomeres	female 209.4 ± 50.6 µm; male 277.3 ± 84 µm	Longitudinal grooves converging towards the tips	Parallel to the antennal surface	Sharp
**SC5**	Dorsal side of the flagellomeres 1st–6th	female 329.4 ± 35.2 µm; male 384.4 ± 82.3 µm	Longitudinal grooves	Shaft diverging from the antennal surface	Tapering
**SC6**	Dorsal side of flagellomeres 1st–5th	female 367 ± 53.2 µm; male 411 ± 68.3 µm	Longitudinal grooves	Shaft irregularly curved	Pointed
**BB**	In groups between the scape and the head and between the scape and the pedicel	female 64.8 ± 17 µm; male 62.5 ± 13 µm	Smooth cuticle	Almost perpendicular to the antennal surface	Sharp
**ST1**	Present only in males on the 1st and 2nd flagellomeres	57.5 ± 4.4 µm	Grooved straight shaft	Parallel to the antennal surface	Pointed
**SB1**	Present in all flagellomeres concentrated in two lateral bands	female 9.5 ± 1 µm; male 8.5 ± 0.7 µm	Curved, smooth	Arising from an elevated base	Blunt-tipped
**SB2**	Present in all flagellomeres concentrated in two lateral bands	female 20.3 ± 3.8 µm; male 18.1 ± 3.3 µm	Thin, bent shaft with smooth surface	Emerging from an elevated socket	Pointed
**SB3**	Few, scattered among SB1 and SB2	female 5.9 ± 0.8 µm; male 5.3 ± 0.8 µm	Smooth at the base, finger like-structures at the tip	Insert into a wide dome	Blunt
**SB4**	1 sensillum ventral side of the 9th flagellomere		Smooth shaft	Raising from a common base	Jointed sensilla with blunt tip

**Table 2:** Antennal secretion hydrocarbons of male and female *A. bungii*.

Peak No.	Hydrocarbon	Percent of Total Hydrocarbons ± S.E. ^1^		Diagnostic Ions
		Male	Female	
1	9:C_23_	N.D. ^2^	1.69 ± 0.37	83, 97, 111 (322)
2	C_25_	1.87 ± 0.25	25.45 ± 5.71	352
3	C_27_-diene	N.D.	4.00 ± 0.66	376
4	2-Me-C_26_	0.54 ± 0.33	21.24 ± 4.43	365, 337 (380)
5	C_27_-monoene	N.D.	10.36 ± 2.25	378
6	C_27_-monoene	N.D.	11.39 ± 1.81	378
7	C_27_	11.27 ± 1.55	9.63 ± 0.96	380
8	C_29_-diene	N.D.	2.41 ± 0.38	404
9	2-Me-C_28_	20.51 ± 4.73	5.62 ± 0.88	365, 393 (408)
10	C_29_-monoene	3.52 ± 0.55	N.D.	406
11	C_29_	15.41 ± 2.88	1.56 ± 0.56	408
12	2-Me-C_28_	3.52 ± 0.31	0.26 ± 0.04	365, 393 (408)
13	2-Me-C_30_	7.11 ± 0.77	6.39 ± 0.61	393, 421 (436)
14	C_31_-monoene	36.24 ± 5.97	N.D.	434

^1^ N = 3 replicates; ^2^ ND = not detected.
